# Two-dimensional binary colloidal crystals formed by particles with two different sizes

**DOI:** 10.1038/s41598-022-16806-y

**Published:** 2022-07-20

**Authors:** Masahide Sato

**Affiliations:** Emerging Media Initiative, Kanazawa University, Kanazawa, 920-1192 Japan

**Keywords:** Materials science, Colloids, Self-assembly

## Abstract

The formation of $\mathrm {AB_2}$ type two-dimensional binary colloidal crystals was studied by performing Monte Carlo simulations with two different size particles. The effect of interactions between particles and between particles and a wall, and the particles size ratios on the formation of $\mathrm {AB_2}$ structure were examined. $\mathrm {AB_2}$ structures formed efficiently when the interaction between equivalently sized particles was smaller than that between differently sized particles. To create $\mathrm {AB_2}$ on a wall, it was necessary to choose a suitable particles size ratios, and the attraction between the particles and the wall was greater than that between particles.

## Introduction

The formation of colloidal crystals has been studied by many groups for the mass-production of fine colloidal crystals because they are promising functional materials for applications such as photonic crystals^1,2^, sensors^3,4^, and catalysis^5,6^. However, understanding how to produce binary colloidal crystals formed by two differently sized particles is required because the properties of binary colloidal crystals can be more easily tuned than those of single-component colloidal crystals by controlling the particle size ratio. With respect to photonic crystals, using patchy particles is one promising way to create a desired structure^7–10^, but binary colloidal crystals are also prominent because the photonic band gap of binary colloidal crystals can be larger than that of single-component colloidal crystals^11^. The Laves phases created by binary colloidal crystals with two differently sized particles are precursors to colloidal crystals with a complete three-dimensional photonic band gap^12–14^. Thus, many groups have studied the formation of binary colloidal crystals^15–19^.

There are three Laves phases with MgCu$_2$, MgZn$_2$, and MgNi$_2$ structures. The diamond and pyrochlore structures, which open up a complete three-dimensional photonic band gap, are obtained with the MgCu$_2$ Laves phase, so that understanding the growth mechanism of these AB$_2$ structures by binary colloidal crystals^14,20^ is necessary to selectively create the MgCu$_2$ Laves phase. Understanding the formation of good quality two-dimensional AB$_2$ colloidal crystals is required because quality three-dimensional functional materials can be created more easily by using the two-dimensional crystals as substrates for epitaxial growth^21–24^ rather than from free growth in an open three-dimensional space. Recently, the formation from solutions of a two-dimensional AB$_2$ crystal on a wall was observed by experiment^25^. The system consisted of particles with two different sizes, and polymers were added for the particles to attract to each other by a depletion force. How the conditions with which the AB$_2$ structures form changed and how this structure grew on the bottom wall were examined by controlling the polymer density, the ratio of the two particle sizes, and the solution compositions.

Considering that experiment^25^, I performed Monte Carlo simulations and studied the formation of $\mathrm {AB_2}$–type binary colloidal crystals on a wall. Herein, the results of the simulations are shown and used to explain how the interaction between particles, the interaction between particles and the bottom wall of a container, the particle size ratio of two types of particles, and the particle density affect the formation of $\mathrm {AB_2}$–type two-dimensional binary crystal. The simulation results are also compared with data obtained by experiment. Two differently sized particles were contained in the system and both the attractive interaction between particles and the interaction between particles and the bottom wall were considered in my simulations. For simplicity, square–well potentials were used as the attractive potentials, even though the attractive interaction between particles may be more complicated in the experiment^25^.

## Results

In the experiment^25^, particles in three-dimensional solutions attached to the bottom wall of a container and two-dimensional AB$_2$ structure was created. So, in my simulations described herein, I focused on how particles which were put into a three-dimensional space form the two-dimensional structure on the wall of a container. Before performing Monte Carlo simulations, I considered which relationships between attractive energies should be satisfied in a two-dimensional system for creating the two-dimensional $\mathrm {AB}_2$ structure. I assumed that the numbers of isolated A and B particles were given by *n* and 2*n* in the two-dimensional system and that the diameter of the A particles, $\sigma _\mathrm {A}$, was larger than that of the B particles, $\sigma _\mathrm {B}$. In the following evaluation, I neglected the effect of particles in the three-dimensional solutions on the particles on the wall. When the A phase and B phase with triangular lattices forms separately, the energy gain by solidification should be given by $-3 n( \varepsilon _\mathrm {BB} + 2 \varepsilon _\mathrm {AA}),$ where $\varepsilon _\mathrm {AA}$, $\varepsilon _\mathrm {BB}$, and $\varepsilon _\mathrm {AB}$ are the bonding energy for A particles, B particles, and A and B particles, respectively. If the A and B particles are well-mixed and $\mathrm {AB}_2$ phase forms, the decrease in the system energy is given by $-6n \varepsilon _{AB}$. For the system with the $\mathrm {AB}_2$ structure to be more stable than that consisting of A and B phases, the relationship between the interaction energies should adhere to the following condition: $(\varepsilon _\mathrm {AA}/2 + \varepsilon _\mathrm {BB})/\varepsilon _\mathrm {AB}<1$. Considering this rough estimation, I performed simulations for $\varepsilon _\mathrm {AA}/\varepsilon _\mathrm {AB} < 1$ and $\varepsilon _\mathrm {BB}/\varepsilon _\mathrm {AB} < 1$, and studied how the formation of the $\mathrm {AB}_2$ structure depends on both $\varepsilon _\mathrm {AA}/\varepsilon _\mathrm {AB}$ and $\varepsilon _\mathrm {BB}/\varepsilon _\mathrm {AB}$. I also studied how this dependence was affected by $\varepsilon _\mathrm {AB}$, the ratio of $\sigma _\mathrm {B}$ to $\sigma _\mathrm {A}$, the strength of the attractive interaction between the bottom wall and the particles $\varepsilon _\mathrm {W}$, and the particle density $\rho$.

### Order parameters for the local twelve-fold rotational symmetry

Simulations were performed in a cubic system. The bottom wall parallel to the *xy*-plane is given by $z=0$. To evaluate how much of the AB$_2$–type structure was created for the particles attaching to the bottom wall, two order parameters were introduced: $\phi _3^\mathrm {A}(m)$ denoting the three-fold rotational order of A particles around *m*th B particles, and $\phi _6^\mathrm {B}(m)$ denoting the six-fold rotational order of B particles around *m*th A particles. The definitions of ${\phi }_3^\mathrm {A}(m)$ and ${\phi }_6^\mathrm {B}(m)$ were given by $\phi _3^\mathrm {A} (m) = n_\mathrm {A}^\mathrm {B} (m)^{-1} \left| \sum _k \mathrm {e}^{3\mathrm {i} \theta _{mk}} \right|$ and $\phi _6^\mathrm {B} (m) = n_\mathrm {B}^\mathrm {A} (m)^{-1} \left| \sum _k \mathrm {e}^{6\mathrm {i} \theta _{mk}} \right|,$ where $n_\mathrm {A}^\mathrm {B} (m)$ and $n_\mathrm {B}^\mathrm {A} (m)$ were the number of the nearest neighbouring A particles around the *m*th B particle and that of the nearest neighbouring B articles around the *i*th A particle, respectively. $\theta _{mk}$ denoted the angle between the *x*-axis and the *xy* plane-component of $\varvec{r}_{ij}=\varvec{r}_i-\varvec{r}_j,$ where $\varvec{r}_i$ was the position of the *i*th particle. Both ${\phi }_3^\mathrm {A}$ and ${\phi }_6^\mathrm {B}$ would approach unity if the AB$_2$–type structure perfectly formed on the bottom wall.

### Dependence of the formation of $\mathrm {AB}_2$**on**$\varepsilon _\mathrm {AA}/\varepsilon _\mathrm {AB}$**and**$\varepsilon _\mathrm {BB}/\varepsilon _\mathrm {AB}$

Simulations were first performed for $\varepsilon _\mathrm {AB}/k_\mathrm {B}T=4.0k_\mathrm {B}T,$ where $k_\mathrm {B}$ was the Boltzmann constant and *T* was the temperature. $\varepsilon _\mathrm {W}$, $\rho$, and $\sigma _\mathrm {B}$ were set to be $2\varepsilon _\mathrm {AB}$, 0.1, and $0.8 \sigma _\mathrm {A}$, respectively.

**Figure 1:**
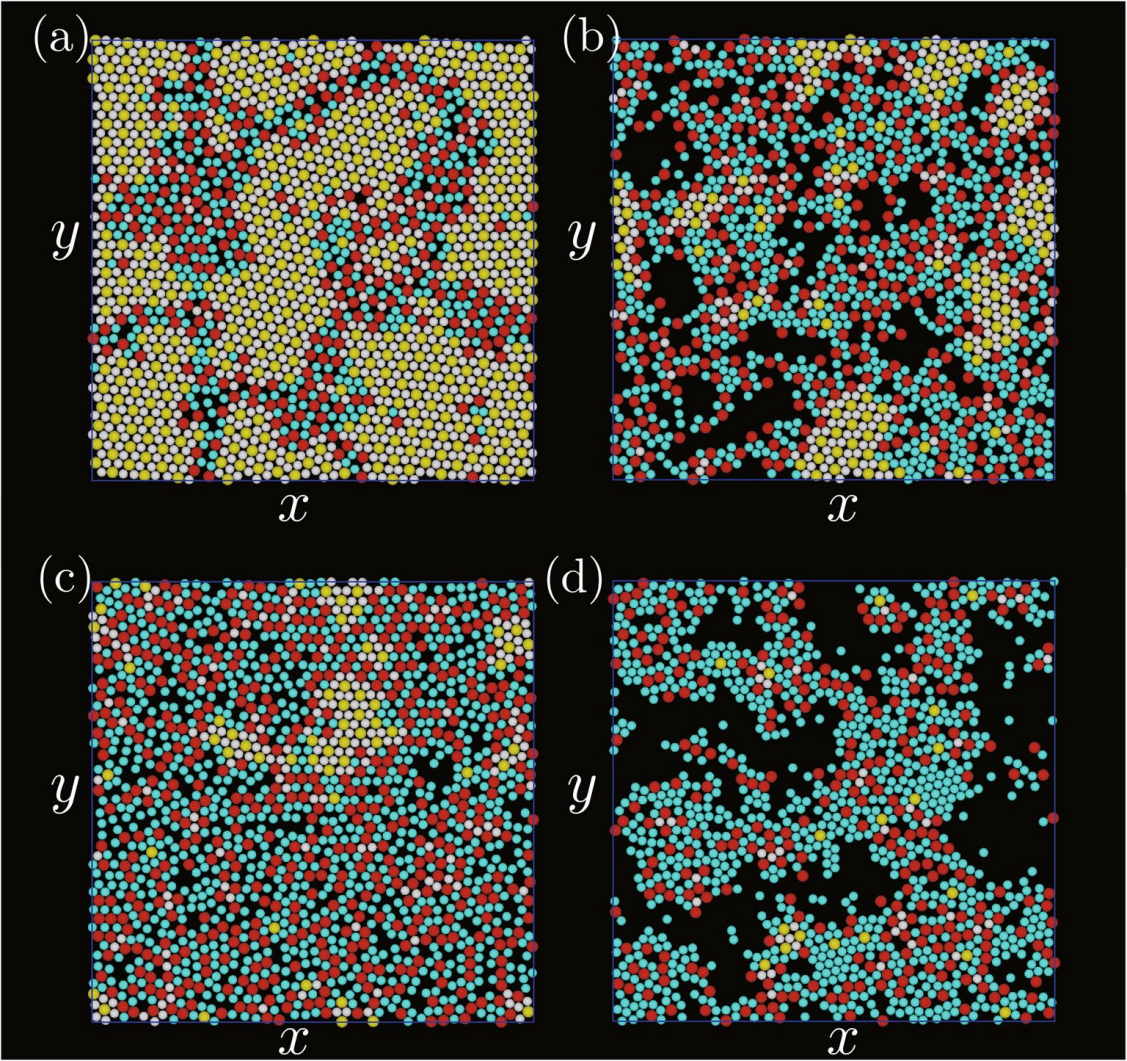
Snapshots of two-dimensional structures created on the bottom wall. $\varepsilon _\mathrm {AA}/\varepsilon _\mathrm {AB}$ and $\varepsilon _\mathrm {BB}/\varepsilon _\mathrm {AB}$ were (**a**) 0.1 and 0.1, (**b**) 0.1 and 0.8, (**c**) 0.8 and 0.1, and (**d**) 0.8 and 0.8. Yellow particles are A particles with $\phi _6^\mathrm {B} >0.7$; white particles are B particles with $\phi _3^\mathrm {A} >0.7$; red particles are A particles with $\phi _6^\mathrm {B} <0.7$; cyan particles are B particles with $\phi _3^\mathrm {A} <0.7$.

**Figure 2:**
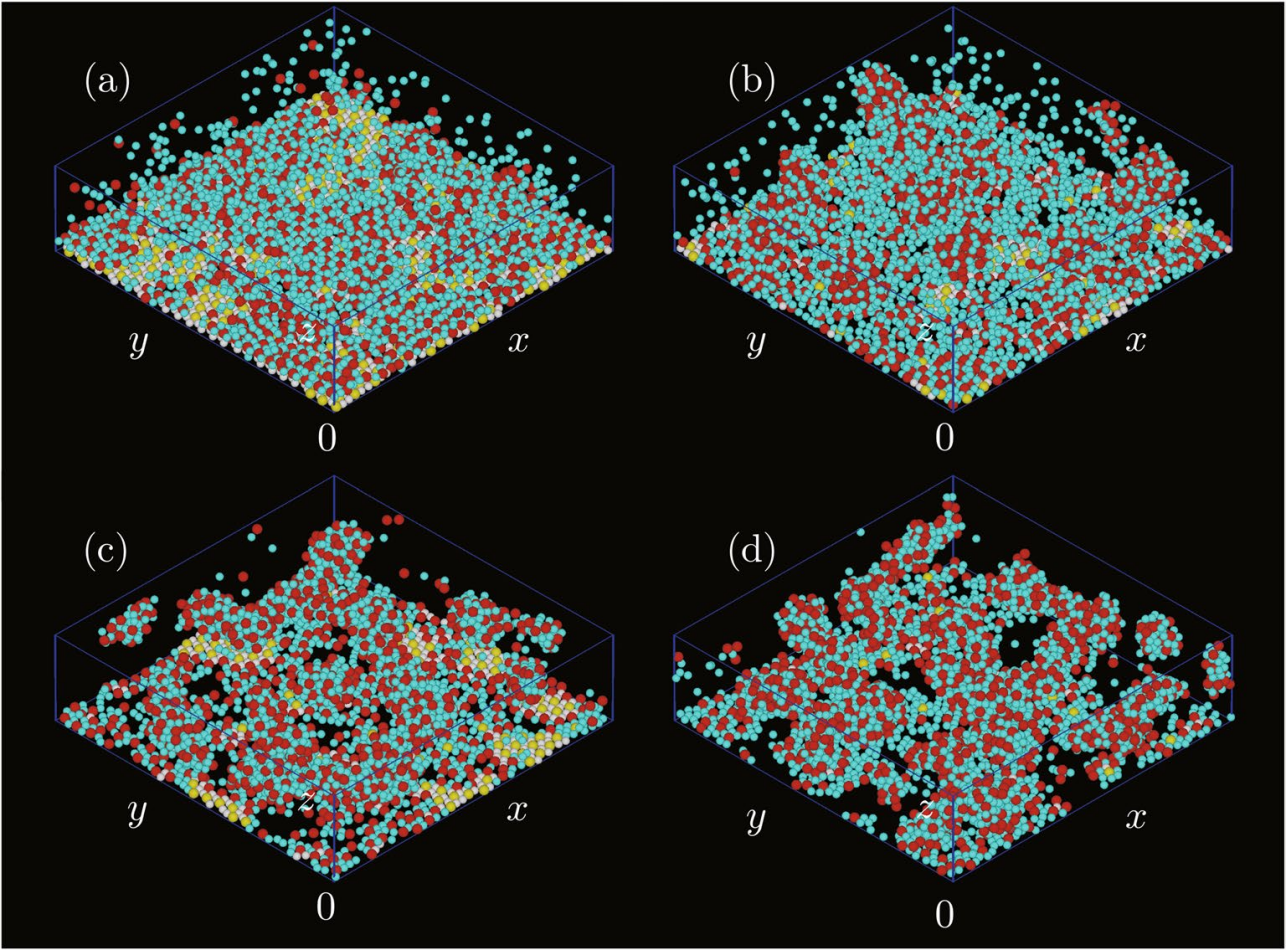
Three-dimensional snapshots of the systems, where $\varepsilon _\mathrm {AA}/\varepsilon _\mathrm {AB}$ and $\varepsilon _\mathrm {BB}/\varepsilon _\mathrm {AB}$ were (**a**) 0.1 and 0.1, (**b**) 0.1 and 0.8, (**c**) 0.8 and 0.1, and (**d**) 0.8 and 0.8. The meanings of the particle colours are the same as those in Fig. 1.

Figure 1 shows snapshots of two-dimensional structures created on the bottom wall for several sets of $\varepsilon _\mathrm {AA}/\varepsilon _\mathrm {AB}$ and $\varepsilon _\mathrm {BB}/\varepsilon _\mathrm {AB}$. The particles with $\phi _3^\mathrm {A}>0.7$ or $\phi _6^\mathrm {B}>0.7$ were regarded as the high local rotational ordered particles, and the local rotational orders were distinguished by particle colours. When both $\varepsilon _\mathrm {AA}/\varepsilon _\mathrm {AB}$ and $\varepsilon _\mathrm {BB}/\varepsilon _\mathrm {AB}$ were small (Fig. 1a), the local rotational order was high for many particles. The number of particles with high rotational order decreased with an increase in either $\varepsilon _\mathrm {AA}/\varepsilon _\mathrm {AB}$ or $\varepsilon _\mathrm {BB}/\varepsilon _\mathrm {AB}$ (Figs. 1b and 1c). The segregation of two types of particles occurred in these two cases because at least one type of particles easily attached to each other owing to the high level of attraction between the same type of particles. In addition to the segregation, the decrease in the number of particles attached to the bottom wall was significant when both $\varepsilon _\mathrm {AA}/\varepsilon _\mathrm {AB}$ and $\varepsilon _\mathrm {BB}/\varepsilon _\mathrm {AB}$ became large (Fig. 1d), which was because the attraction between particles was sufficiently high enough for particles to form three-dimensional clusters. While the particles solidified two-dimensionally on the bottom wall when both $\varepsilon _\mathrm {AA}/\varepsilon _\mathrm {AB}$ and $\varepsilon _\mathrm {BB}/\varepsilon _\mathrm {AB}$ were small (Fig. 2a), the shape of the solid became more three-dimensional when at least one of the energy ratios became high as in Fig. 2b, and three-dimensional clusters were observed clearly when both energy ratios were high as in Fig. 2c and 2d.

**Figure 3:**
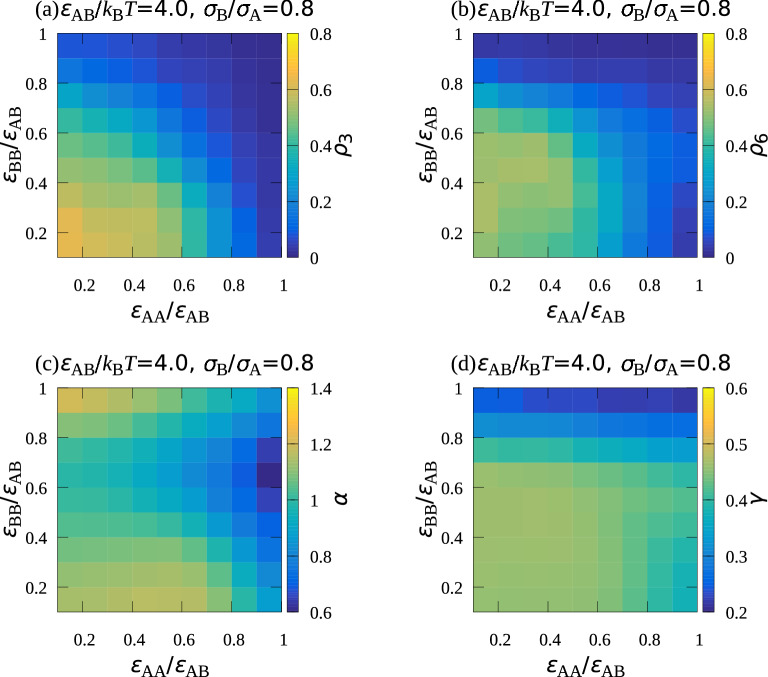
Dependence of (**a**) $\rho _3$, (**b**) $\rho _6$, (**c**) $\alpha$, and (**d**) $\gamma$ on $\varepsilon _\mathrm {AA}/\varepsilon _\mathrm {AB}$ and $\varepsilon _\mathrm {BB}/\varepsilon _\mathrm {AB},$ where $\varepsilon _\mathrm {AB}/k_\mathrm {B}T=4.0$, $\sigma _\mathrm {B}/\sigma _\mathrm {A}=0.8$, and $\rho =0.1$. $\rho _3$ and $\rho _6$ represent the ratios of highly ordered B particle and A particle to all the particles attached to the bottom wall, respectively, $\alpha$ shows how the composition ratio of A particles on the bottom wall is higher than that in the system, and $\gamma$ shows the ratio of the number of particles attaching to the wall to that of all the particles in the system.

The dependence of the highly ordered particle densities on the energies were also examined. Figs. 3a and 3b, show how the ratio of highly ordered B particles, $\rho _\mathrm {3}$, and that of the A particles, $\rho _\mathrm {6}$, to all the particles attached to the bottom wall depend on $\varepsilon _\mathrm {AA}/\varepsilon _\mathrm {AB}$ and $\varepsilon _\mathrm {BB}/\varepsilon _\mathrm {AB}$, respectively. Both $\rho _3$ and $\rho _6$ decreased with an increase in either $\varepsilon _\mathrm {AA}/\varepsilon _\mathrm {AB}$ or $\varepsilon _\mathrm {BB}/\varepsilon _\mathrm {AB}$ because, as already mentioned, the segregation of the A and B particles occurred when the level of attractive interaction between the same type of particles was high. Compared with the region with large $\rho _3$, the region with large $\rho _6$ was shifted slightly toward the large $\varepsilon _\mathrm {BB}/\varepsilon _\mathrm {AB}$ direction. Figure 3c shows $\alpha =n_\mathrm {A}/(n_\mathrm {A}+n_\mathrm {B}) /[N_\mathrm {A}/(N_\mathrm {A}+N_\mathrm {B})],$ where $N_\mathrm {A}$ and $N_\mathrm {B}$ represents the number of A particles and B particle in the system, respectively, and $n_\mathrm {A}$ and $n_\mathrm {B}$ represents those on the wall, respectively. $\alpha$ showed how the ratio of the number of A particles to all the particle attaching to the wall was larger than the ratio of the number of A particles to all the particles put in the system. $\alpha$ was large when both $\varepsilon _\mathrm {AA}/\varepsilon _\mathrm {AB}$ and $\varepsilon _\mathrm {BB}/\varepsilon _\mathrm {AB}$ were small. In this energy region, because ratio of the number of A particles to that of B particles in the system was set to be 0.3 in the simulation, A particles were in surplus on the bottom wall after creating the two-dimensional AB$_2$ structure. Thus, $\rho _6$ became small owing to the surplus A particles when both $\varepsilon _\mathrm {AA}/\varepsilon _\mathrm {AB}$ and $\varepsilon _\mathrm {BB}/\varepsilon _\mathrm {AB}$ were small, and large $\rho _6$ region was shifted slightly.

Also studied was how the ratio of the number of particles attaching to the bottom wall to the total particle number, $\gamma =( n_\mathrm {A} + n_\mathrm {B})/(N_\mathrm {A}+N_\mathrm {B} )$, depended on $\varepsilon _\mathrm {AA}/\varepsilon _\mathrm {AB}$ and $\varepsilon _\mathrm {BB}/\varepsilon _\mathrm {AB},$ where $N_\mathrm {A}$ and $N_\mathrm {B}$ were the numbers of A and B particles put in the system, respectively (Fig. 3d). $\gamma >0.5$ in almost the entire region. Large $\gamma$ seems natural because, considering that $\varepsilon _\mathrm {W}$ is larger than $\varepsilon _\mathrm {AB}$, particles preferentially attached to the bottom wall. However, $\gamma$ decreases and $\gamma < 0.5$ when $\varepsilon _\mathrm {BB}$ was large, which might be because of the strong attraction between B particles, which were much contained in the system, as they aggregated in the three-dimensional space rather than becoming attached to the bottom wall. In the simulations, the collision of two A particles must have been less frequent than that of two B particles because $N_\mathrm {A} < N_\mathrm {B}$. That means the decrease in the number of particles attaching to the bottom walls became greater with an increasse in $\varepsilon _\mathrm {BB}$ rather than with an increase in $\varepsilon _\mathrm {AA}$.

### Effects of $\sigma _\mathrm {B}/\sigma _\mathrm {A}$**on the formation of AB**$_2$**structure**

To examine how $\sigma _\mathrm {B}/\sigma _\mathrm {A}$ affects the two-dimensional structure formed on the bottom wall, simulations with different $\sigma _\mathrm {B}$ were performed. Figures S1 and S2 show snapshots of two-dimensional structures on the bottom wall for $\sigma _\mathrm {B}/\sigma _\mathrm {A}=0.85$ and $\sigma _\mathrm {B}/\sigma _\mathrm {A}=0.7$, respectively. Figures S3 and S4 show how $\varepsilon _\mathrm {AA}/\varepsilon _\mathrm {AB}$ and $\varepsilon _\mathrm {BB}/\varepsilon _\mathrm {AB}$ affect $\rho _3$, $\rho _6$, $\alpha$, and $\gamma$ for these $\sigma _\mathrm {B}/\sigma _\mathrm {A}$. From the figures it can be seen that the dependence of them on $\varepsilon _\mathrm {AA}/\varepsilon _\mathrm {AB}$ and $\varepsilon _\mathrm {BB}/\varepsilon _\mathrm {AB}$ was qualitatively the same as for $\sigma _\mathrm {B}/\sigma _\mathrm {A}=0.8$.

**Figure 4:**
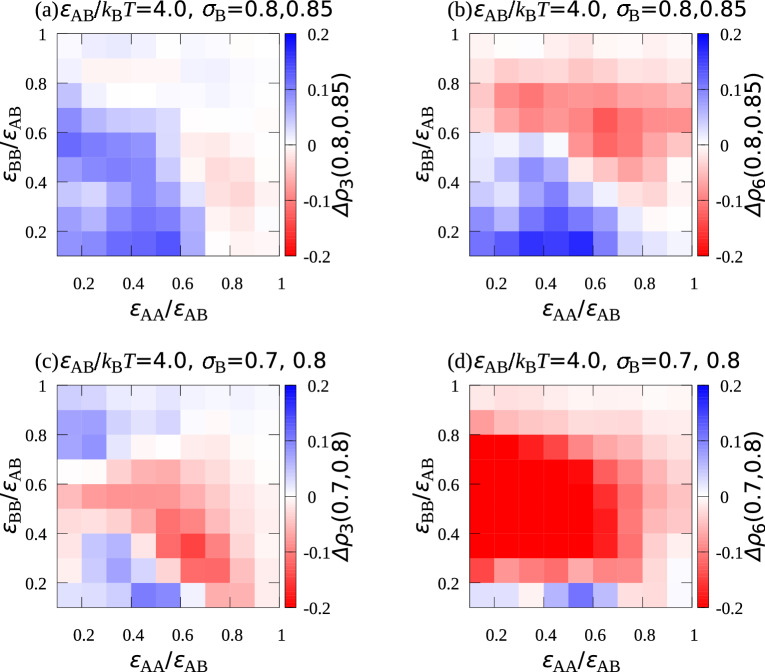
Difference between (**a**) $\rho _3$ in the system with $\sigma _\mathrm {B}=0.8$ and that in the system with $\sigma _\mathrm {B}=0.85$, (**b**) $\rho _6$ in the system with $\sigma _\mathrm {B}=0.8$ and that in the system with $\sigma _\mathrm {B}=0.85$, (**c**) $\rho _3$ in the system with $\sigma _\mathrm {B}=0.7$ and that in the system with $\sigma _\mathrm {B}=0.8$, and (**d**) $\rho _6$ in the system with $\sigma _\mathrm {B}=0.7$ and that in the system $\sigma _\mathrm {B}=0.8,$ where $\sigma _\mathrm {A}$ was set to unity.

The changes in $\rho _3$ and $\rho _6$ for $\sigma _\mathrm {B}/\sigma _\mathrm {A}=$ 0.7, 0.8, and 0.85 were examined in more detail. Figure 4a shows $\Delta \rho _3(0.8, 0.85)=\rho _3({\sigma _\mathrm {B}/\sigma _\mathrm {A}=0.8})-\rho _3({\sigma _\mathrm {B}/\sigma _\mathrm {A}=0.85})$. $\Delta \rho _3(0.8, 0.85)$ was positive for the region with small $\varepsilon _\mathrm {AA}/\varepsilon _\mathrm {AB}$ and small $\varepsilon _\mathrm {BB}/\varepsilon _\mathrm {AB},$ where the AB$_2$ structure formed. Because $\Delta \rho _6(0.8, 0.85)$ was also positive in the region, as shown in Fig. 4b, $\sigma _\mathrm {B}/\sigma _\mathrm {A}=0.8$ was more suitable than $\sigma _\mathrm {B}/\sigma _\mathrm {A}=0.85$ for creating the AB$_2$ structure. $\Delta \rho _6(0.7, 0.8)$ was negative in almost the region with small $\varepsilon _\mathrm {AA}/\varepsilon _\mathrm {AB}$ and small $\varepsilon _\mathrm {BB}/\varepsilon _\mathrm {AB}$ (Fig. 4d). For $\Delta \rho _3(0.8, 0.85)$ (Fig. 4c), the negative $\Delta \rho _3(0.8, 0.85)$ region seemed to start spreading from around $\varepsilon _\mathrm {BB}/\varepsilon _\mathrm {AB} =0.5$ towards the region with small $\varepsilon _\mathrm {BB}/\varepsilon _\mathrm {AB}$. Thus, these results showed that the most suitable $\sigma _\mathrm {B}/\sigma _\mathrm {A}$ existed at about $\sigma _\mathrm {B}/\sigma _\mathrm {A}=0.8$ for creating the AB$_2$ structure.

### Effects of $\varepsilon _\mathrm {W}$**on the formation of the AB**$_2$**structure**

It is natural that $\varepsilon _\mathrm {W}$ affects the formation of the AB$_2$ structure because in the simulations, this structure was created on the bottom wall by particles attaching to the wall. In the simulations discussed until now, $\varepsilon _\mathrm {W}$ was set to $2\varepsilon _\mathrm {AB}$. Simulations were also performed simulations with other $\varepsilon _\mathrm {W}$ to examine how $\varepsilon _\mathrm {W}$ affects the formation of the AB$_2$ structure.

**Figure 5:**
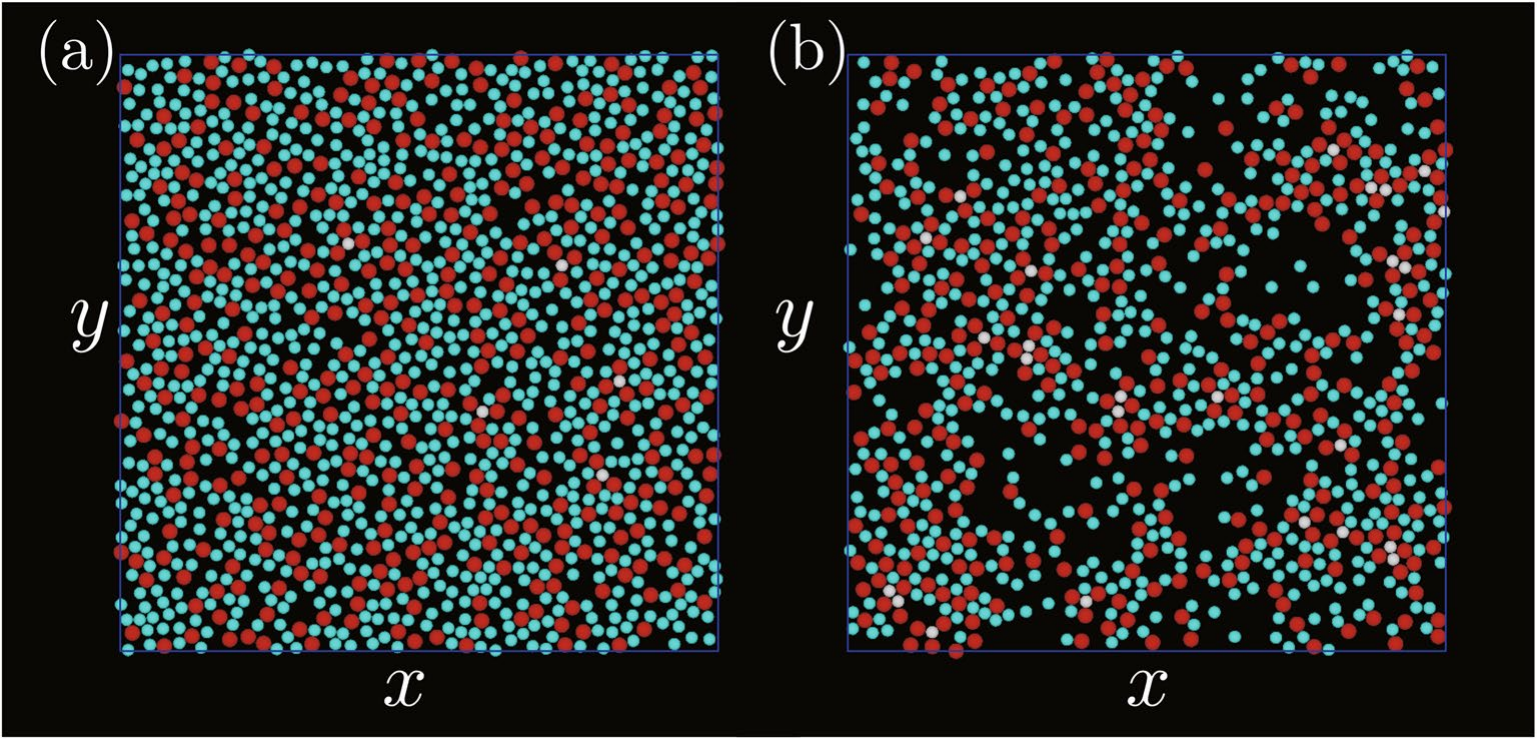
Snapshots of two-dimensional structure created on the bottom wall for (**a**) $\varepsilon _\mathrm {W}=4\varepsilon _\mathrm {AB}$ and (**b**) $\varepsilon _\mathrm {AB},$ where he energy ratios $\varepsilon _\mathrm {AA}/\varepsilon _\mathrm {AB}$ and $\varepsilon _\mathrm {BB}/\varepsilon _\mathrm {AB}$ were both set to 0.1, and $\varepsilon _\mathrm {AB}/k_\mathrm {B}T$ was the same as that in Fig. 1.

Figure 5a and b show snapshots for $\varepsilon _\mathrm {W}=4\varepsilon _\mathrm {AB}$ and $\varepsilon _\mathrm {W}=\varepsilon _\mathrm {AB}$, respectively. Clearly, $\rho _3$ and $\rho _6$ in both cases were smaller than those for $\varepsilon _\mathrm {W}=2\varepsilon _\mathrm {AB}$ (Fig. 1). Figures S5 and S6 show detailed analyses. Compared with Fig. S5d, the number of particles attaching to the bottom wall increased with an increase in $\varepsilon _\mathrm {W}$ (Fig. S6d). However, $\rho _6$ and $\rho _3$ were both low in the entire parameter region and few ordered particles were observed for both $\varepsilon _\mathrm {W}=4\varepsilon _\mathrm {AB}$ and $\varepsilon _\mathrm {AB}$ (Figs. S5a, b, S6a and b), which means that there is a suitable $\varepsilon _\mathrm {W}$ for creating the AB$_2$ structure on the bottom wall. $\alpha$ was different between Figs. S5c and S6c. For $\varepsilon _\mathrm {W}=\varepsilon _\mathrm {AB}$, $\alpha$ was small when $\varepsilon _\mathrm {AA}/\varepsilon _\mathrm {AB}$ was large and $\varepsilon _\mathrm {BB}/\varepsilon _\mathrm {AB}$ was small, because the A particles preferred to aggregate three-dimensionally rather than to attach to the bottom wall. This was because of the strong attractive interaction between A particles (Fig. S5c). However, $\alpha$ in this parameter region became large when $\varepsilon _\mathrm {W}=4\varepsilon _\mathrm {AB}$ because $\varepsilon _\mathrm {W}$ was so large that the A particles preferred to attach to the bottom wall rather than to aggregate three-dimensionally.

### Effects of $\varepsilon _\mathrm {AB}$**on the formation of AB**$_2$**structure**

**Figure 6:**
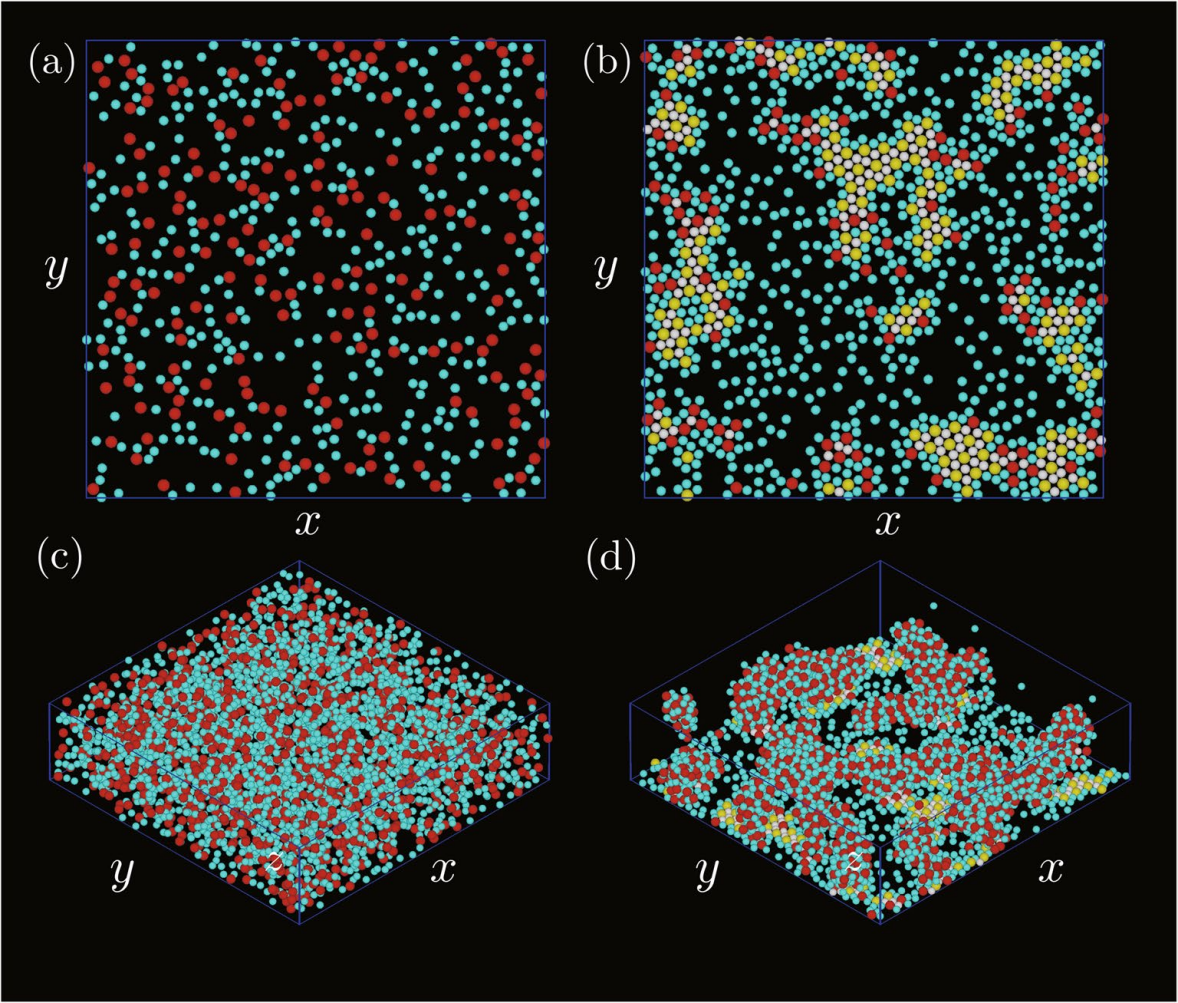
Two-dimensional structures created on the bottom wall for $\varepsilon _\mathrm {AB}/k_\mathrm {B}T=$ (**a**) 2.0 and (**b**) 6.0, and three-dimensional views for $\varepsilon _\mathrm {AB}/k_\mathrm {B}T=$ (**c**) 2.0 and (**d**) 6.0. The strength of interactions were $\varepsilon _\mathrm {AA}/\varepsilon _\mathrm {AB}=\varepsilon _\mathrm {BB}/\varepsilon _\mathrm {AB}=0.1$.

In the above analyses, the effects of $\varepsilon _\mathrm {AA}/\varepsilon _\mathrm {AB}$ and $\varepsilon _\mathrm {BB}/\varepsilon _\mathrm {AB}$ on the formation of AB$_2$ structure were studied, but how the absolute value of $\varepsilon _\mathrm {AB}$ affected to the two-dimensional structure was not examined . I therefore examined the effects of $\varepsilon _\mathrm {AB}$ on the formation of the AB$_2$ structure, while the two ratios $\varepsilon _\mathrm {AA}/\varepsilon _\mathrm {AB}$ and $\varepsilon _\mathrm {BB}/\varepsilon _\mathrm {AB}$ were the same as those in the above simulations. Figure 6 shows snapshots for $\varepsilon _\mathrm {AB}/k_\mathrm {B}T=2.0$ (Figs. 6a and 6c) and $\varepsilon _\mathrm {AB}/k_\mathrm {B}T=6.0$ (Fig. 6b and 6d). For $\varepsilon _\mathrm {AB}/k_\mathrm {B}T=2.0$, the interactions between particles were so weak that no tight bonding of particles formed. The particles seemed to be uniformly distributed in the three-dimensional space (Fig. 6c). However, for $\varepsilon _\mathrm {AB}/k_\mathrm {B}T=6.0$, particles were bonded tightly but $\varepsilon _\mathrm {AB}$ was so large that the particles aggregated three-dimensionally, as in Fig. 2d. Not only did the number of particles attaching to the bottom wall decrease, but the particles on the bottom wall did not show high rotational order. Thus, only few particles with high rotational order appeared on the bottom wall in both cases.

### Particle density effects

In the simulations, the ordering of particles on the bottom wall was changed by the attractive interaction between particles. When the attraction between particles was strong, the solid formed three-dimensionally. If the particle density was higher, the collision of particles occurred more frequently. Thus, clusters were created more easily in the three-dimensional space, causing a decrease in the two-dimensional ordering of particles on the bottom wall. I expect that ordering of particles on the bottom walls may also be affected by the width of the layers of particles piled in the *z*-direction. i Finally, to examine the effect of particle density on the two-dimensional ordering of particles on the bottom wall, simulations were performed with a higher particle density.

**Figure 7:**
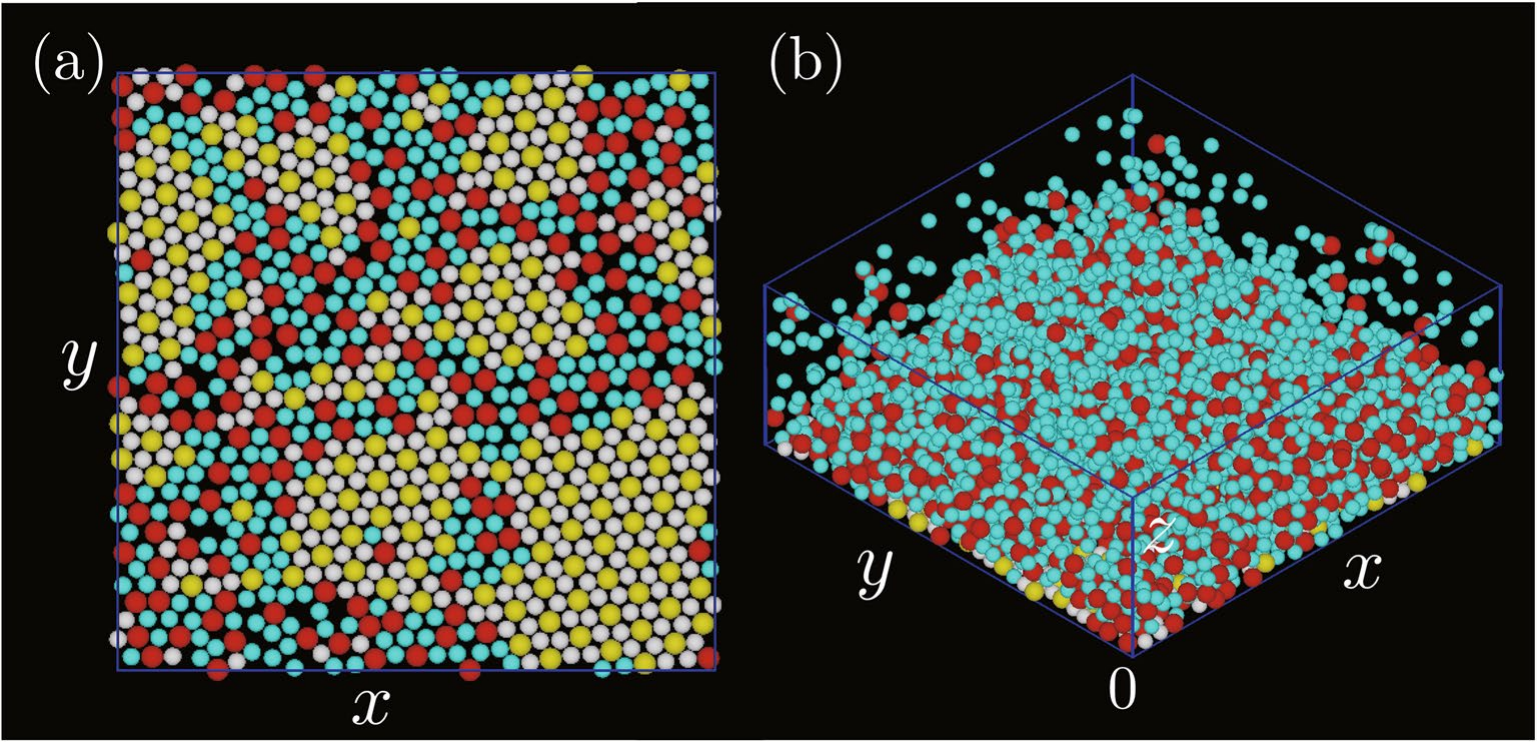
(**a**) Two-dimensional structure on the bottom wall and (**b**) Three-dimensional view for $\varepsilon _\mathrm {AA}/\varepsilon _\mathrm {AB}=\varepsilon _\mathrm {BB}/\varepsilon _\mathrm {AB}=0.1$ for $\rho =0.2$.

**Figure 8:**
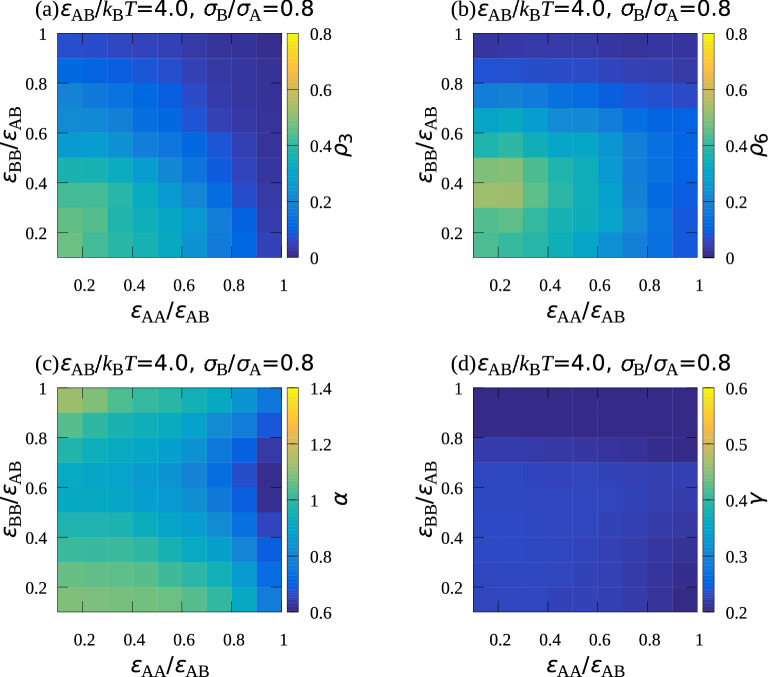
Dependence of (**a**) $\rho _3$, (**b**) $\rho _6$, (**c**) $\alpha$, and (**d**) $\gamma$ on $\varepsilon _\mathrm {AA}/\varepsilon _\mathrm {AB}$ and $\varepsilon _\mathrm {BB}/\varepsilon _\mathrm {AB},$where $\varepsilon _\mathrm {AB}/k_\mathrm {B}T=4.0$, $\sigma _\mathrm {B}/\sigma _\mathrm {A}=0.8$, and $\rho =0.2$. $\rho _3$ and $\rho _6$ represent the ratios of highly ordered B particle and A particle to all the particles attached to the bottom wall, respectively, $\alpha$ shows how the composition ratio of A particles on the bottom wall is higher than that in the system, and $\gamma$ shows the ratio of the number of particles attaching to the wall to that of all the particles in the system.

Figure 7a and b show snapshots of the bottom wall and a birds eye view for $\varepsilon _\mathrm {AA}/\varepsilon _\mathrm {AB}=0.1$ and $\varepsilon _\mathrm {BB}/\varepsilon _\mathrm {AB}=0.1$, respectively. The particle density, $\rho$, was 0.2, which is twice as large as that in the simulations described thus far. Compared with Fig. 2a, many particles were deposited on the first layer. From Fig. 7a, the particles attaching to the bottom wall seemed to have as high a rotational order as those for $\rho =0.1$ (Fig. 1a). Figure 8 showed the dependence of $\rho _3$, $\rho _6$, $\alpha$, and $\gamma$ on the energies for $\rho =0.2$. $\gamma$ was much smaller than that for $\rho =0.1$, which was reasonable because the area of the bottom wall became small with the increase in $\rho$ (Fig. 8d). $\alpha$ seemed to be similar to that for $\rho =0.1$ (Fig. 8c). $\rho _3$ and $\rho _6$ (Figs. 8a and 8b). also showed the same tendency although they were a little smaller than those for $\rho =0.1$. Although the decline in the ordering of particles on the bottom wall was small, two-dimensional ordering of particles on the bottom wall decreased with the increase in the particle density, which is probably because the three-dimensional connection between particles disturbed the orientation of the plane with the AB$_2$ structure on the bottom wall.

## Summary and discussions

Here, my simulation results are summarized and compared with previous studies. In Fig. S7, how the typical structure depended on $\varepsilon _\mathrm {AA}/\varepsilon _\mathrm {AB}$ and $\varepsilon _\mathrm {BB}/\varepsilon _\mathrm {AB}$ is shown. In all the cases, AB$_2$ structure formed when both $\varepsilon _\mathrm {AA}/\varepsilon _\mathrm {AB}$ and $\varepsilon _\mathrm {BB}/\varepsilon _\mathrm {AB}$ were small. I also found that there was a suitable value of $\sigma _\mathrm {B}/\sigma _\mathrm {A}$ for the formation of AB$_2$ structure from Figs. S7a–c, and it was difficult to create AB$_2$ structure when the particle density was large from Fig. S7a and d.

As shown in the supplementary, $\varepsilon _\mathrm {AA}/\varepsilon _\mathrm {AB}$ for the depletion potential should be larger than unity. In an experiment^25^, it was reported that the ratio of energy values to the temperature is smaller than that in our simulations. Thus, unfortunately, the relationship between energies obtained our simulations for creating AB$_2$ structure are not realized by the depletion effect, which was considered to be the main effect in an experiment^25^. However, the interactions I used in my simulations may be feasible for other systems such as the system with DNA-coated colloids^26–30^ because similar ratios of the interaction strength to the temperature, with which the AB$_2$ structure was obtained in my simulations, was realized in an experiment^29^, and the interaction length and strength can be deliberately designed for DNA-coated colloids using DNA strands as the source of interaction between particles.

My simulation results showed that the attractive interaction between particles and the wall needed to be greater than that between particles for the AB$_2$ structure to be created on the wall. In a previous experiment^24^, in agreement with my simulations, the nucleation of colloidal crystals occurred first on a flat bottom wall during sedimentation, and the interaction between particles and the flat surface mainly affected the spontaneous nucleation. As shown in the supplementary, the interaction between particles and the wall are roughly twice as large as that between particles. Thus, even if the interaction between particles I considered in the simulations are realized in other systems, the attractive interaction between particles and the wall is also necessary.

## Model

### System setting

Canonical Monte Carlo simulations were performed in a cubic system whose size was given by $L_x \times L _y\times L_z$. Periodic boundary conditions were used in the *x*- and *y*- directions and walls were considered in the *z*-directions. Large A particles of diameter $\sigma _\mathrm {A}$ and small B particles of diameter $\sigma _\mathrm {B}$ were put into the system. The total number of particles, $N= N_\mathrm {A}+ N_\mathrm {B}$, was set to 4096, and the composition ratio of A particles $f=N_\mathrm {A}/(N_\mathrm {A}+N_\mathrm {B})$, was set to 0.3 because I had expected that $\mathrm {AB_2}$-type crystals would be well formed when *f* is equaled the ratio of B to A in the $\mathrm {AB}_2$ crystal. $\sigma _\mathrm {A}$ was set to unity and simulations were performed for several $\sigma _\mathrm {B}$. The distance between two walls at $z=0$ and $L_z$ was set to 10. When the particle density $\rho$ was set, the system lengths $L_x$ and $L_y$ were given by $L_x=L_y= [\pi ( \sigma _\mathrm {B}^3 N_\mathrm {B}+ \sigma _\mathrm {A}^3 N_\mathrm {A} )/(6 L_z\rho ) ]^{1/2}$. Particles were put into the system at random. and $10^7$ Monte Carlo trials were performed for each particle. To avoid too small acceptance rate in the trials, the maximum amplitude of translation was tuned every 100 Monte Carlo trials.

### Interaction between particles and interaction between a wall and particles

A square-well potential was used as the attractive interaction potential between particles. The potential $U(r_{ij})$ for the *i*th and *j*th particles was given by

1 $$\begin{aligned} U(r_{ij}) = {\left\{ \begin{array}{ll} \infty &{}(r_{ij}< \sigma _{ij}) \\ -\varepsilon _{ij} &{} (\sigma _{ij} \le r_{ij} \le \sigma _{ij} + \Delta ) \\ 0 &{} (\sigma _{ij} + \Delta < r_{ij}) \end{array}\right. }, \end{aligned}$$

where $\varvec{r}_i$ represented the position of the centre of the *i*th particle, and $r_{ij}=|\varvec{r}_j-\varvec{r}_i|$. The attractive length, $\Delta$, was set to be $5 \times 10^{-2}$ regardless of the type of interacting particles, but $\sigma _{ij}$ and $\varepsilon _{ij}$ depended on the particle type: $\sigma _{ij}$ and $\varepsilon _{ij}$ were $\sigma _\mathrm {B}$ and $\varepsilon _\mathrm {BB}$, $\sigma _\mathrm {A}$ and $\sigma _\mathrm {AA}$, and $(\sigma _\mathrm {A}+\sigma _\mathrm {B})/2$ and $\varepsilon _\mathrm {AB}$ for B particles, A particles, and A and B particles, respectively. I also considered the interaction between particles and the bottom wall placed at $z=0$. The interaction potential between the wall and particles, $U^\mathrm {W}(z_i)$, was assumed to be a square-well potential given by

2 $$\begin{aligned} U^\mathrm {W}(z_{i})={\left\{ \begin{array}{ll} \infty &{}(z_{i}< \sigma _{i}) \\ -\varepsilon _\mathrm {W} &{} \left( \displaystyle \frac{\sigma _{i}}{2} \le z_{i} \le \frac{\sigma _{i}}{2} + \Delta \right) \\ 0 &{} (\sigma _{i} + \Delta < z_{i}) \end{array}\right. }, \end{aligned}$$

where $z_i$ was the *z*-coordinate of the *i*th particle, and $\sigma _{i}$ was $\sigma _\mathrm {A}$ for A particles and $\sigma _\mathrm {B}$ for B particles.

## Supplementary Information

Supplementary Information.

## Data Availability

The datasets used and analysed during the current study available from the corresponding author on reasonable request.
