# Supplementary material for: Two-dimensional binary colloidal crystals formed by particles with two different sizes
Source: Sci Rep. 2022 Jul 20;12:12370. doi: 10.1038/s41598-022-16806-y (PMC9300637; doi:10.1038/s41598-022-16806-y)
Supplement: Supplementary file 1 — Supplementary Information. [file 41598_2022_16806_MOESM1_ESM.pdf]

Supplymentary Information: Two-Dimensional Binary Colloidal Crystals Formed by Particles with Two Different Sizes

Masahide, Sato[1,*]

[1]Emerging Media Initiative, Kanazawa University, Kanazawa 920-1192, Japan
[*]msato002@staff.kanazawa-u.ac.jp

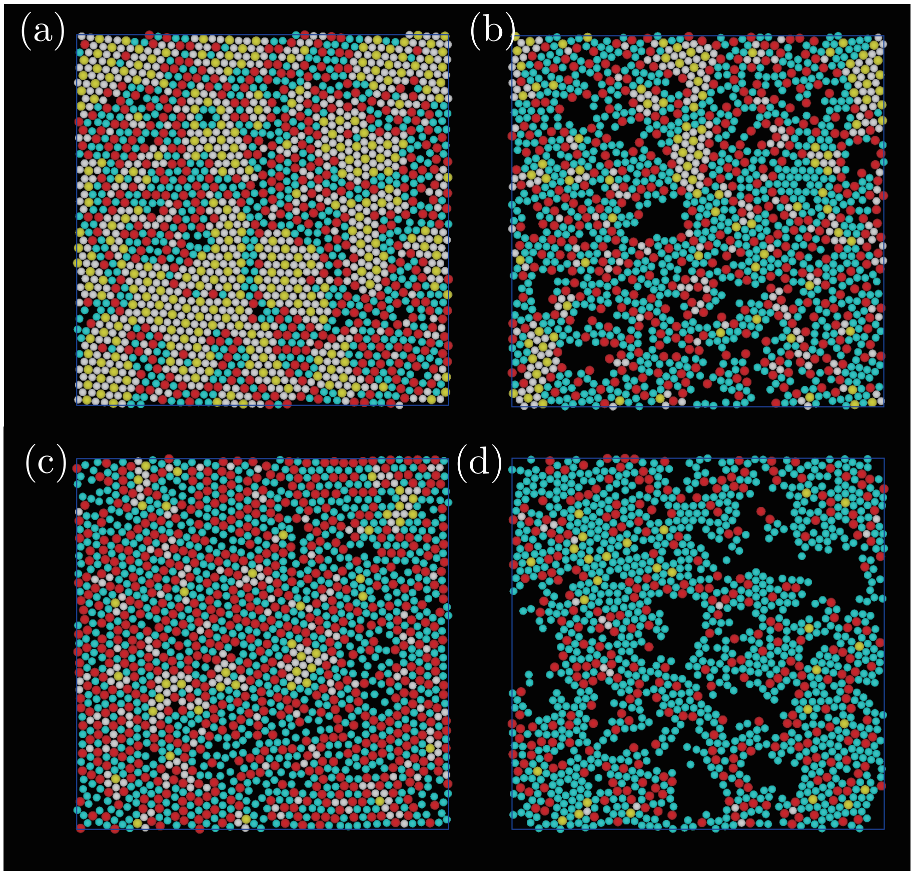

Figure S1. Snapshots of two-dimensional structure created on the bottom wall for $\sigma_{\mathrm{B}}/\sigma_{\mathrm{A}} = 0.85$. $\varepsilon_{\mathrm{AA}}/\varepsilon_{\mathrm{AB}}$ and $\varepsilon_{\mathrm{BB}}/\varepsilon_{\mathrm{AB}}$ were (a) 0.1 and 0.1, (b) 0.1 and 0.8, (c) 0.8 and 0.1, and (d) 0.8 and 0.8. Yellow particles are A particles with $\phi_6^{\mathrm{B}} > 0.7$; white particles are B particles with $\phi_3^{\mathrm{A}} > 0.7$; red particles are A particles with $\phi_6^{\mathrm{B}} < 0.7$; cyan particles are B particles with $\phi_3^{\mathrm{A}} < 0.7$.

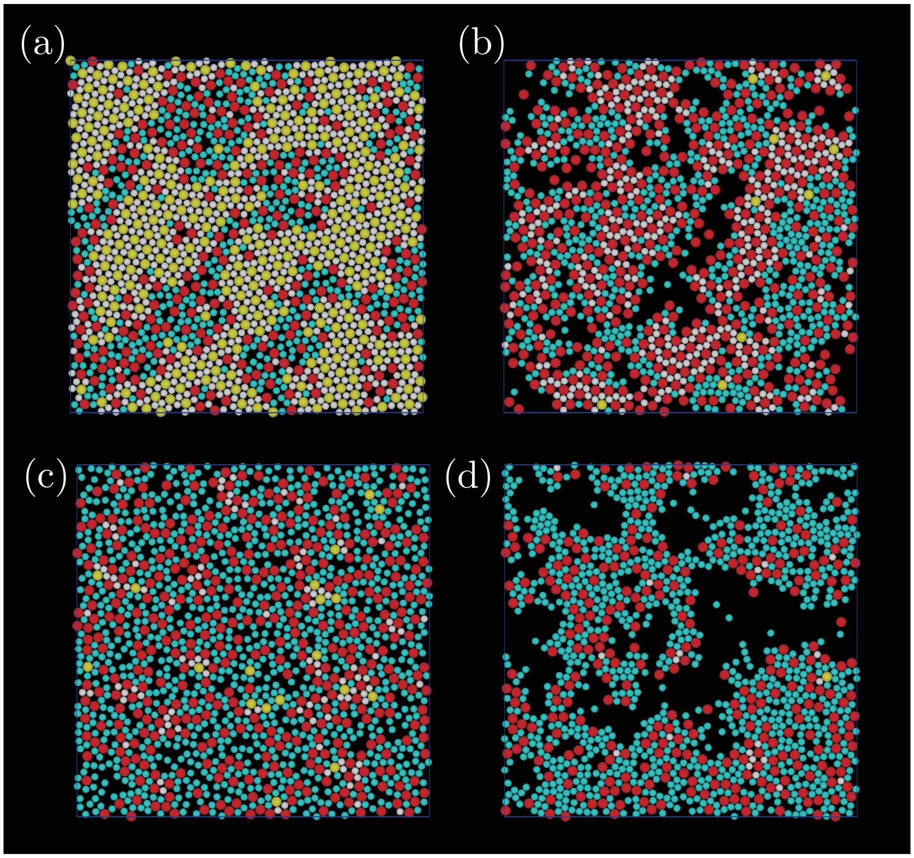

Figure S2. Snapshots of two-dimensional structure created on the bottom wall placed at $z = 0$. for $\sigma_B/\sigma_A = 0.70$. $\varepsilon_{AA}/\varepsilon_{AB}$ and $\varepsilon_{BB}/\varepsilon_{AB}$ were (a) 0.1 and 0.1, (b) 0.1 and 0.8, (c) 0.8 and 0.1, and (d) 0.8 and 0.8. The meaning of colours are the same as that in Fig. S1.

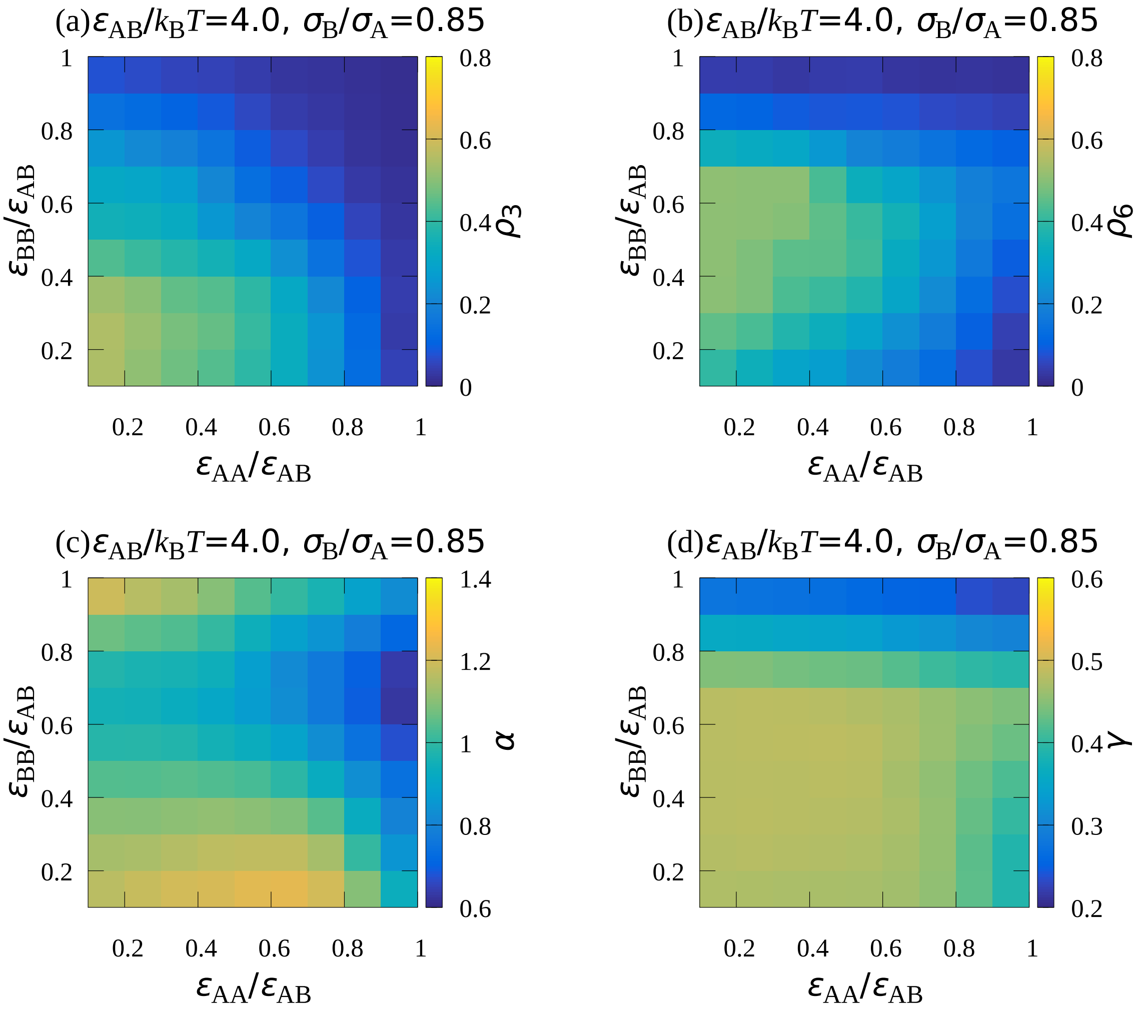

Figure S3. Dependence of (a)ϕ_3, (b)ϕ_6, (c)α, and (d)γ on $\varepsilon_{AA}/\varepsilon_{AB}$ and $\varepsilon_{BB}/\varepsilon_{AB}$, where $\varepsilon_{AB}/k_B T = 4.0$, $\sigma_B/\sigma_A = 0.85$, $\rho = 0.1$, and $\varepsilon_w = 2\varepsilon_{AB}$. ρ_3 and ρ_6 represent the ratios of highly ordered B particle and A particle to all the particles attached to the bottom wall, respectively, α shows how the composition ratio of A particles on the bottom wall is higher than that in the system, and γ shows the ratio of the number of particles attaching to the wall to that of all the particles in the system.

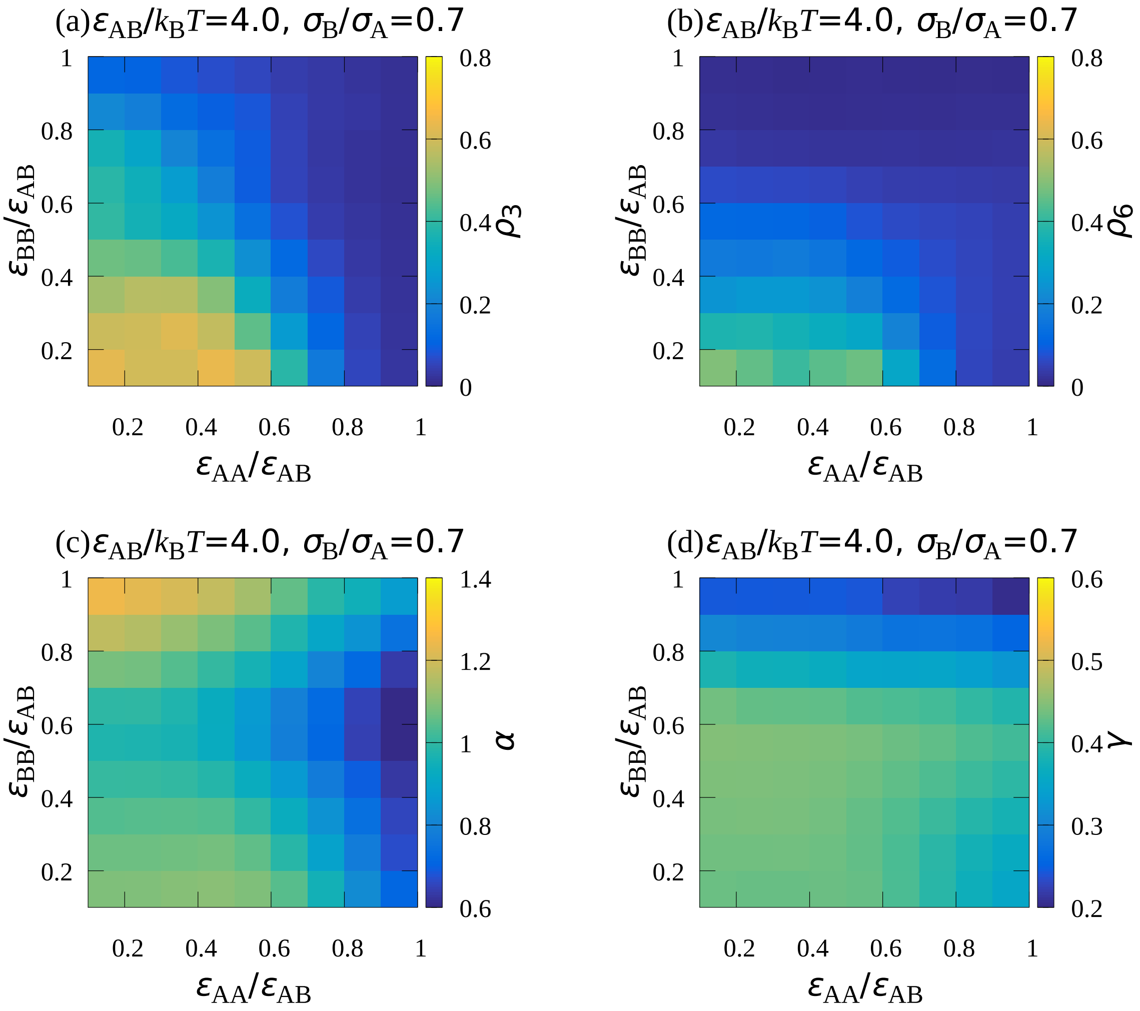

Figure S4. Dependence of (a)ϕ_3, (b)ϕ_6, (c)α, and (d)γ on $\varepsilon_{AA}/\varepsilon_{AB}$ and $\varepsilon_{BB}/\varepsilon_{AB}$, where $\varepsilon_{AB}/k_BT = 4.0$, $\sigma_B/\sigma_A = 0.7$, $\rho = 0.1$, and $\varepsilon_W = 2\varepsilon_{AB}$. ρ_3 and ρ_6 represent the ratios of highly ordered B particle and A particle to all the particles attached to the bottom wall, respectively, α shows how the composition ratio of A particles on the bottom wall is higher than that in the system, and γ shows the ratio of the number of particles attaching to the wall to that of all the particles in the system.

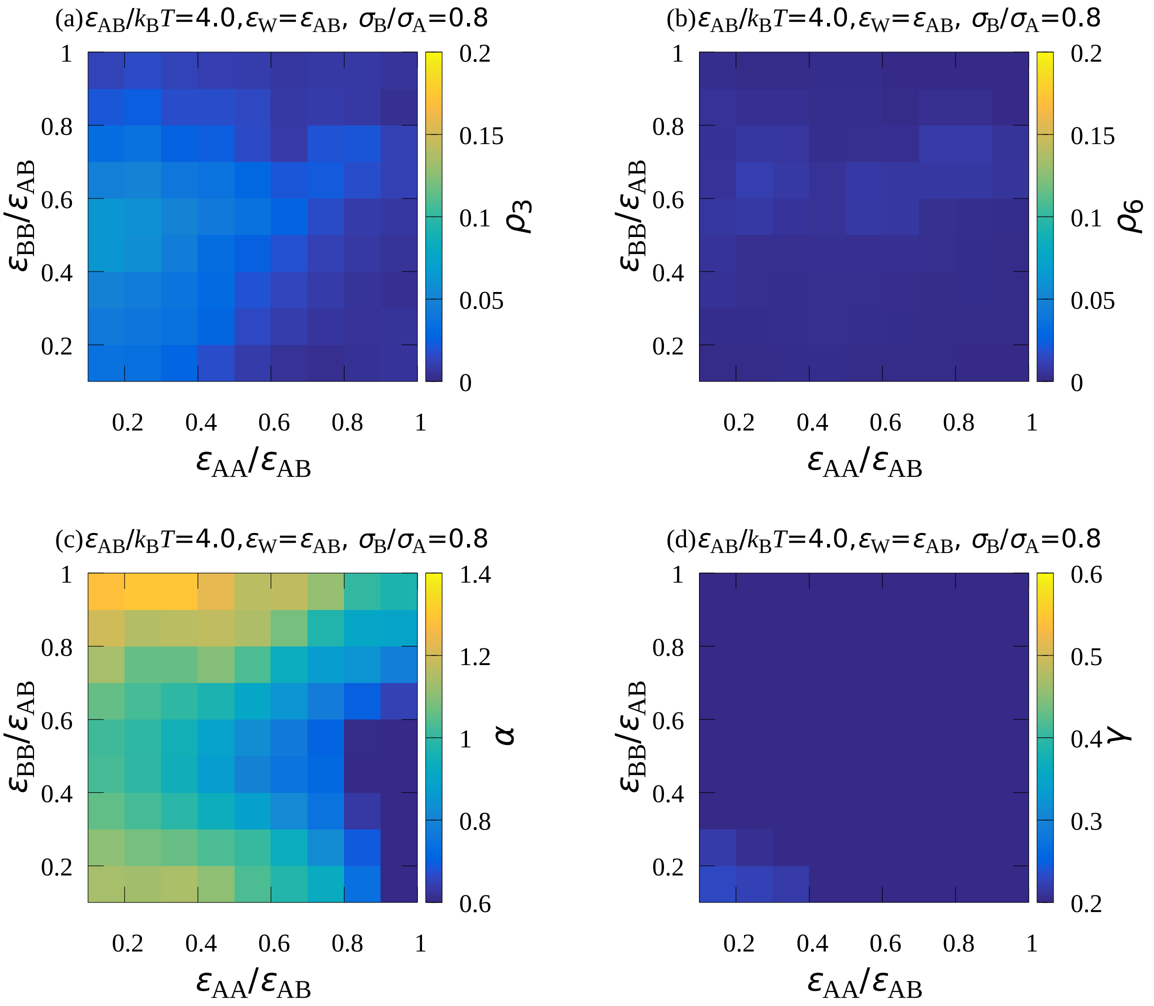

Figure S5. Dependence of (a)ϕ_3, (b)ϕ_6, (c)α, and (d)γ on $\varepsilon_{AA}/\varepsilon_{AB}$ and $\varepsilon_{BB}/\varepsilon_{AB}$, where $\varepsilon_{AB}/k_BT = 2.0$, $\sigma_B/\sigma_A = 0.8$, $\rho = 0.1$, and $\varepsilon_W = \varepsilon_{AB}$. ρ_3 and ρ_6 represent the ratios of highly ordered B particle and A particle to all the particles attached to the bottom wall, respectively, α shows how the composition ratio of A particles on the bottom wall is higher than that in the system, and γ shows the ratio of the number of particles attaching to the wall to that of all the particles in the system.

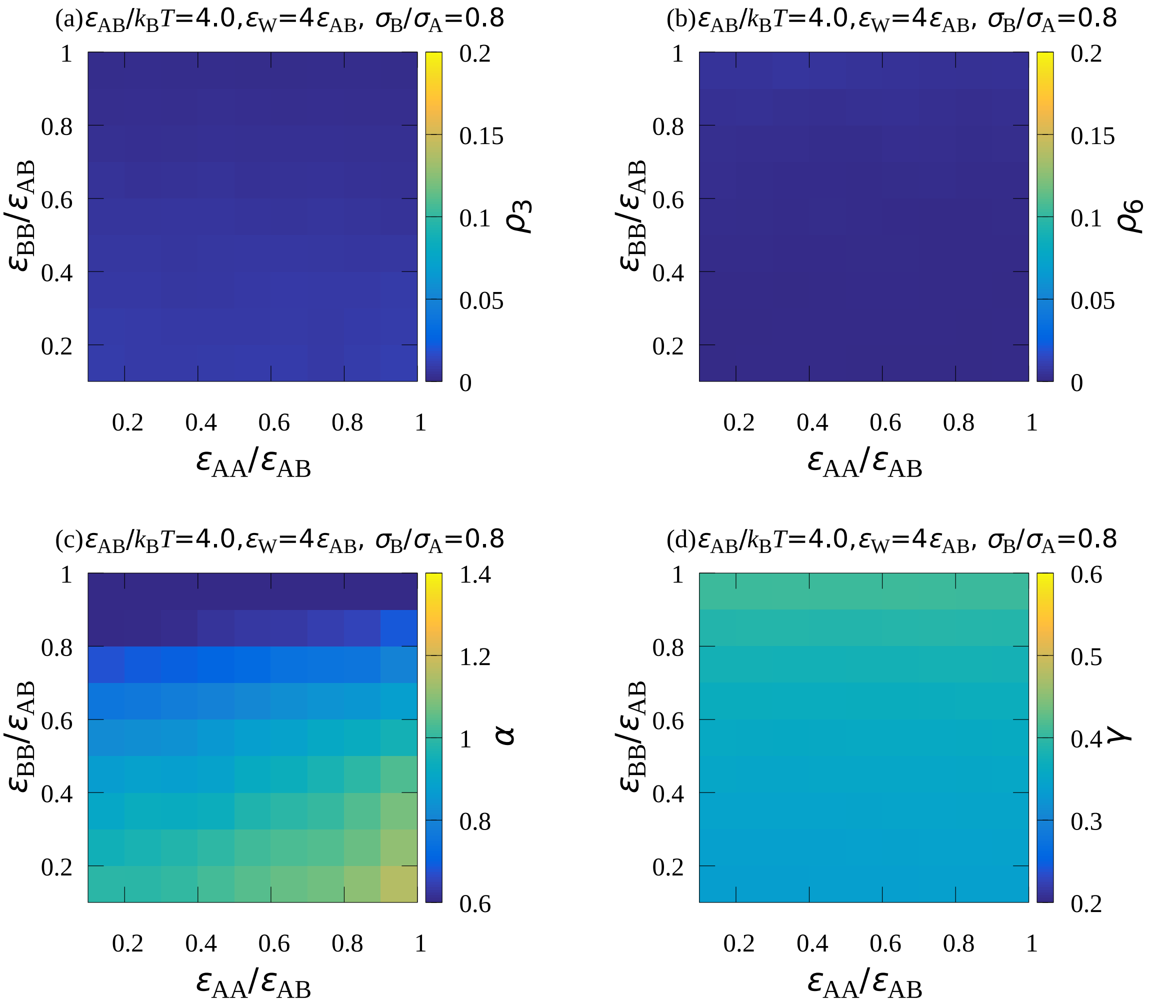

Figure S6. Dependence of (a)ϕ_3, (b)ϕ_6, (c)α, and (d)γ on $\varepsilon_{AA}/\varepsilon_{AB}$ and $\varepsilon_{BB}/\varepsilon_{AB}$, where $\varepsilon_{AB}/k_BT = 6.0$, $\sigma_B/\sigma_A = 0.8$, $\rho = 0.1$, and $\varepsilon_W = 4\varepsilon_{AB}$. ρ_3 and ρ_6 represent the ratios of highly ordered B particle and A particle to all the particles attached to the bottom wall, respectively, α shows how the composition ratio of A particles on the bottom wall is higher than that in the system, and γ shows the ratio of the number of particles attaching to the wall to that of all the particles in the system.

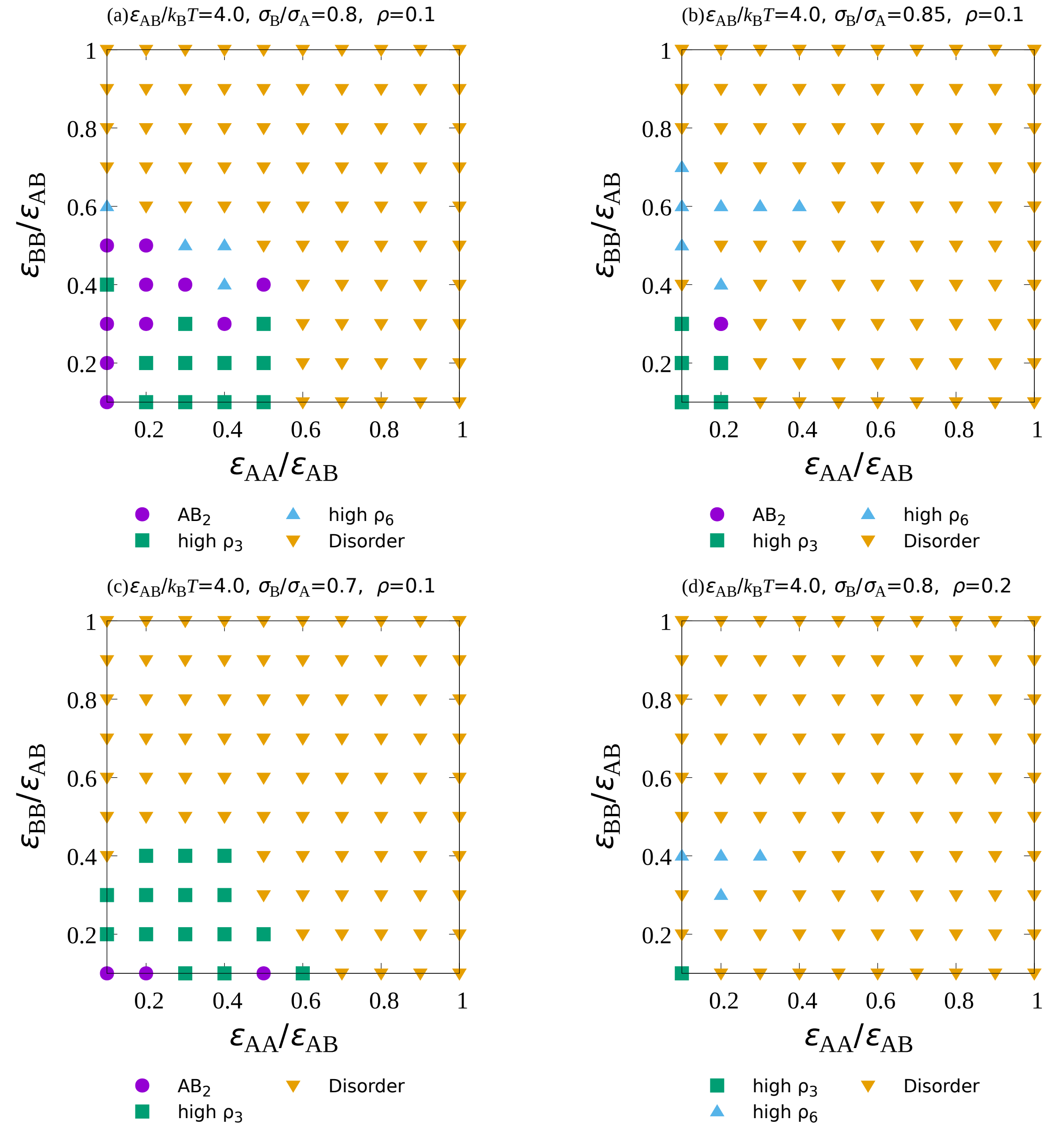

Figure S7. Dominant structures for (a) $\sigma_B/\sigma_A = 0.8$ and $\rho = 0.1$, (b) $\sigma_B/\sigma_A = 0.85$ and $\rho = 0.1$, (c) $\sigma_B/\sigma_A = 0.7$ and $\rho = 0.1$, and (d) $\sigma_B/\sigma_A = 0.7$ and $\rho = 0.2$. Circles show the parameters with $\rho_3 > 0.5$ and $\rho_6 > 0.5$; squares show the parameters with $\rho_3 > 0.5$ and $\rho_6 < 0.5$; triangles show the parameters with $\rho_3 < 0.5$ and $\rho_6 > 0.5$; inversed triangles show the parameters with $\rho_3 < 0.5$ and $\rho_6 < 0.5$. In all the figures, ε_{AB}/k_BT was set to 4.0.

Possibility of the depletion effect as attraction

I consider a particle with the radius r_x and that with radius r_y. The interaction potential between the two particles induced by the depletion attraction, $U_{\mathrm{OA}}(r)$ is given by

$$U_{\mathrm{OA}} = \begin{cases} -n_p k_{\mathrm{B}} T V_{\mathrm{OV}}(r) & (2r_d < r < 2R_d) \\ 0 & (r > 2R_d) \end{cases}, \tag{1}$$

where r is the distance between the centers of two particles and n_p is the overlap concentration of polymer. $r_d = (r_x + r_y)/2$ and $R_d = \sigma_d + R_g$, where R_g is the radius of gyration of polymer. $V_{\mathrm{OV}}(r)$ is the overlap volume of spheres whose radii are $r_x + R_g$ and $r_y + R_g$. When the two particles attach to each other, the overlap volume $V_{\mathrm{OV}}(2r_d)$ is given by

$$V_{\mathrm{OV}}(2r_d) = \frac{\pi(r_x+R_g)^3}{3}(1-\cos\theta_x)^2(2+\cos\theta_x) + \frac{\pi(r_y+R_g)^3}{3}(1-\cos\theta_y)^2(2+\cos\theta_y), \tag{2}$$

where $\cos\theta_x$ and $\cos\theta_y$ are given by

$$\cos\theta_x = \frac{(r_x+R_g)^2-(r_y+R_g)^2+(r_x+r_y)^2}{2(r_x+r_y)(r_x+R_g)}, \tag{3}$$

$$\cos\theta_y = \frac{(r_y+R_g)^2-(r_x+R_g)^2+(r_x+r_y)^2}{2(r_x+r_y)(r_y+R_g)}. \tag{4}$$

If R_g is much smaller than r_x and r_y, $\cos\theta_x$ and $\cos\theta_y$ are approximated as

$$\cos\theta_x = \left(1 + \frac{(r_x-r_y)}{r_x(r_x+r_y)}R_g - \frac{1}{r_x}R_g\right), \tag{5}$$

$$\cos\theta_y = \left(1 + \frac{(r_y-r_x)}{r_y(r_x+r_y)}R_g - \frac{1}{r_y}R_g\right). \tag{6}$$

Thus, the interaction energy is given by $4\pi R_g^2 r_x r_y/(r_x+r_y)$. When I regard $n_p k_{\mathrm{B}} T V_{\mathrm{OV}}(2r_d)$ as the interaction energy between particles, $\varepsilon_{\mathrm{AA}}$, $\varepsilon_{\mathrm{BB}}$ and $\varepsilon_{\mathrm{AB}}$ are given by $\pi n_p k_{\mathrm{B}} T R_g^2 \sigma_{\mathrm{A}}$, $\pi n_p k_{\mathrm{B}} T R_g^2 \sigma_{\mathrm{B}}$, and $2\pi n_p k_{\mathrm{B}} T R_g^2 \sigma_{\mathrm{A}}\sigma_{\mathrm{B}}/(\sigma_{\mathrm{A}}+\sigma_{\mathrm{B}})$, respectively. Thus, $\varepsilon_{\mathrm{AA}}/\varepsilon_{\mathrm{AB}}$ is given by $(\varepsilon_{\mathrm{AA}}+\varepsilon_{\mathrm{AB}})/2\varepsilon_{\mathrm{AB}}$ and always larger than unity.

The attraction between a particle and a wall is obtained when one particle radius is set to be a constance and the other radius approaches infinity. The attraction energy between a particle with radius $\sigma/2$ and a wall is given by $\pi n_p k_{\mathrm{B}} T R_g^2 \sigma$, which is twice as large as the attractive energy between two particles with radius $R/2$.
